# Supplementary material for: COZOID: contact zone identifier for visual analysis of protein-protein interactions
Source: BMC Bioinformatics. 2018 Apr 6;19:125. doi: 10.1186/s12859-018-2113-6 (PMC5889581; doi:10.1186/s12859-018-2113-6)
Supplement: Supplementary file 2 — Software build. Executable binary file of the software tool. (ZIP 118,784 kb) [file 12859_2018_2113_MOESM2_ESM.zip › cozoid/platform/docs/testng-6.5.1-javadoc.zip!/constant-values.html]

Constant Field Values (TestNG)


---


|  |  |  |
| --- | --- | --- |
| **Overview** \| Package \| Class \| **Tree** \| **Deprecated** \| **Index** \| **Help** | |  |
| PREV   NEXT | **FRAMES**    **NO FRAMES**     **All Classes** |  |


---


# Constant Field Values


---

**Contents**

- org.testng.\*

org.testng.\*

| org.testng.CommandLineArgs | | |
| --- | --- | --- |
| `public static final java.lang.String` | `CONFIG_FAILURE_POLICY` | `"-configfailurepolicy"` |
| `public static final java.lang.String` | `DATA_PROVIDER_THREAD_COUNT` | `"-dataproviderthreadcount"` |
| `public static final java.lang.String` | `DEBUG` | `"-debug"` |
| `public static final java.lang.String` | `EXCLUDED_GROUPS` | `"-excludegroups"` |
| `public static final java.lang.String` | `GROUPS` | `"-groups"` |
| `public static final java.lang.String` | `HOST` | `"-host"` |
| `public static final java.lang.String` | `JUNIT` | `"-junit"` |
| `public static final java.lang.String` | `LISTENER` | `"-listener"` |
| `public static final java.lang.String` | `LOG` | `"-log"` |
| `public static final java.lang.String` | `MASTER` | `"-master"` |
| `public static final java.lang.String` | `METHOD_SELECTORS` | `"-methodselectors"` |
| `public static final java.lang.String` | `METHODS` | `"-methods"` |
| `public static final java.lang.String` | `MIXED` | `"-mixed"` |
| `public static final java.lang.String` | `OBJECT_FACTORY` | `"-objectfactory"` |
| `public static final java.lang.String` | `OUTPUT_DIRECTORY` | `"-d"` |
| `public static final java.lang.String` | `PARALLEL` | `"-parallel"` |
| `public static final java.lang.String` | `PORT` | `"-port"` |
| `public static final java.lang.String` | `RANDOMIZE_SUITES` | `"-randomizesuites"` |
| `public static final java.lang.String` | `REPORTER` | `"-reporter"` |
| `public static final java.lang.String` | `SKIP_FAILED_INVOCATION_COUNTS` | `"-skipfailedinvocationcounts"` |
| `public static final java.lang.String` | `SLAVE` | `"-slave"` |
| `public static final java.lang.String` | `SUITE_NAME` | `"-suitename"` |
| `public static final java.lang.String` | `SUITE_THREAD_POOL_SIZE` | `"-suitethreadpoolsize"` |
| `public static final java.lang.String` | `TEST_CLASS` | `"-testclass"` |
| `public static final java.lang.String` | `TEST_JAR` | `"-testjar"` |
| `public static final java.lang.String` | `TEST_NAME` | `"-testname"` |
| `public static final java.lang.String` | `TEST_NAMES` | `"-testnames"` |
| `public static final java.lang.String` | `TEST_RUNNER_FACTORY` | `"-testrunfactory"` |
| `public static final java.lang.String` | `THREAD_COUNT` | `"-threadcount"` |
| `public static final java.lang.String` | `USE_DEFAULT_LISTENERS` | `"-usedefaultlisteners"` |
| `public static final java.lang.String` | `VERBOSE` | `"-verbose"` |
| `public static final java.lang.String` | `XML_PATH_IN_JAR` | `"-xmlpathinjar"` |
| `public static final java.lang.String` | `XML_PATH_IN_JAR_DEFAULT` | `"testng.xml"` |

| org.testng.ITestResult | | |
| --- | --- | --- |
| `public static final int` | `FAILURE` | `2` |
| `public static final int` | `SKIP` | `3` |
| `public static final int` | `STARTED` | `16` |
| `public static final int` | `SUCCESS` | `1` |
| `public static final int` | `SUCCESS_PERCENTAGE_FAILURE` | `4` |

| org.testng.TestNG | | |
| --- | --- | --- |
| `public static final java.lang.String` | `DEFAULT_COMMAND_LINE_SUITE_NAME` | `"Command line suite"` |
| `public static final java.lang.String` | `DEFAULT_COMMAND_LINE_TEST_NAME` | `"Command line test"` |
| `public static final java.lang.String` | `DEFAULT_OUTPUTDIR` | `"test-output"` |
| `protected static final int` | `HAS_FAILURE` | `1` |
| `protected static final int` | `HAS_FSP` | `4` |
| `protected static final int` | `HAS_NO_TEST` | `8` |
| `protected static final int` | `HAS_SKIPPED` | `2` |
| `public static final java.lang.String` | `SHOW_TESTNG_STACK_FRAMES` | `"testng.show.stack.frames"` |
| `public static final java.lang.String` | `TEST_CLASSPATH` | `"testng.test.classpath"` |

| org.testng.xml.XmlSuite | | |
| --- | --- | --- |
| `public static final java.lang.String` | `CONTINUE` | `"continue"` |
| `public static final java.lang.String` | `PARALLEL_CLASSES` | `"classes"` |
| `public static final java.lang.String` | `PARALLEL_INSTANCES` | `"instances"` |
| `public static final java.lang.String` | `PARALLEL_METHODS` | `"methods"` |
| `public static final java.lang.String` | `PARALLEL_NONE` | `"none"` |
| `public static final java.lang.String` | `PARALLEL_TESTS` | `"tests"` |
| `public static final java.lang.String` | `SKIP` | `"skip"` |

---


|  |  |  |
| --- | --- | --- |
| **Overview** \| Package \| Class \| **Tree** \| **Deprecated** \| **Index** \| **Help** | |  |
| PREV   NEXT | **FRAMES**    **NO FRAMES**     **All Classes** |  |


---
